# The Role of Ipsilateral Tonsillectomy in the Extirpation of Branchial Cleft Anomalies- A Retrospective Monocentric Analysis Over 13 Years

**DOI:** 10.1007/s12070-023-03543-5

**Published:** 2023-04-21

**Authors:** Lukas S. Fiedler, Lorenz F. Fiedler

**Affiliations:** 1 Department of Otorhinolaryngology & Head & Neck Surgery, University Medical Center Hamburg-Eppendorf, Martinistraße 52, 20251 Hamburg, Germany; 2 Medical University of Graz, Auenbruggerplatz 2, 8036 Graz, Styria, Austria

**Keywords:** Branchial cleft anomalies, Tonsillectomy, Relapse, Recurrence

## Abstract

**Backround:**

Branchial cleft anomalies (BCA) can occur as sinuses, fistulas or cysts. They arise from the first, second, third or fourth pharyngeal cleft due to non-fusion or subinvolution. Mostly, located in Robbin’s neck-level II, BCA clinically present as a painless compressible swelling, cutaneous draining sinus, or fistula.

**Aims:**

Surgical treatment is the gold standard to prevent recurrence in BCA, though the necessity of ipsilateral tonsillectomy is discussed and was being examined within this work.

**Methods:**

In retrospect, data was collected from patients, that were admitted with the diagnosis BCA between 2006 and 2020 in an academic tertiary care center. 160 patients met inclusion criteria, the data was further evaluated, the focus was set on the occurrence of recurrence.

**Results:**

Recurrence of BCA was observed in 2 out of 160 surgically treated patients (1,25%), one of them with simultaneous tonsillectomy, the other without.

**Conclusion:**

A statistically significant difference in the recurrence-rate between these two groups (with/without tonsillectomy) could not be shown. The performance of an ipsilateral simultaneous tonsillectomy in the surgical workup of BCA cannot be recommended at the basis of our data.

**Supplementary Information:**

The online version contains supplementary material available at 10.1007/s12070-023-03543-5.

## Introduction

During the embryological development, in the fourth week of gestation, six pairs of arches, clefts and pouches form the branchial or pharyngeal apparatus. Every arch consists of a cartilaginous element, muscular component, a corresponding branch of the aortic arch and a cranial nerve. These components later form various structures in the head and neck and due to non-fusion or subinvolution can result in branchial cleft anomalies (BCA) [1, 2]. These BCA can occur as sinuses, fistulas or cysts and are present at birth, although maybe symptomatic until later in adulthood [2, 3]. BCA comprise about 20% of congenital lesions in children and arise from the first, second, third or fourth pharyngeal cleft [2, 4].

Whereas first branchial cleft anomalies can be divided into Work type I (preauricular and lateral to the facial nerve) and Work type II (mandibular angle/submandibular and medial/or lateral to the facial nerve), third branchial anomalies present in the middle and lower third of the sternocleidomastoid muscle (SCM). The fourth BCA are extremely rare (1%) and normally present in the middle portion of the SCM [2].

BCA arising from the second pharyngeal cleft are the most common and represent 40–95% [2, 5]. They are usually located in the lateral neck anterior and medial to the SCM and can have contact to the ipsilateral pharyngeal, explicitly the tonsillar region [2, 5, 6]. The majority of BCA present as cysts between the age of 20–40, in younger age (< 5 years) sinuses and fistulas are more common [5].

### Clinical Presentation of BCA

Clinically, the majority of BCA present as a painless compressible swelling, draining sinuses, or fistulae situated at the anterior border of SCM in line between the mandibular angle and clavicle [7]. Presenting patients may report a variety of duration and periods of waxing and waning of the neck swelling. Acute size increase can occur due to upper respiratory tract infections [5]. Secondary infections and inflammation can occur, therefore neck abscesses are possible [8].

Although rare, bilateral second branchial cleft cysts have been reported [9] and in some patients this is part of the branchio-oto-renal syndrome (BOR), an autosomal dominant disorder [10]. BOR or Melnick-Fraser-Syndrome [11] symptoms include hearing impairment, cup-shaped pinnae, preauricular pits, branchial fistulae and renal anomalies [5]. Even though there is a positive predictive value in preoperative diagnosis of BCA, cystic neck masses should presumed malignant [12]. In this cases, fine needle aspiration (FNAC) can bring light into the differentiation of the cystic mass and identify malignant tumours [13], especially in lymphoma an important tool to prevent from pretherapeutic surgery.

### Treatment of BCA

To effectively treat second, third and fourth cleft BCA, total surgical excision is recommended [14–17]. A strict differentiation between sinuses, cysts or fistulae is necessary to guarantee the optimal choice of surgical technique and approach [16]. Within the treatment of fistulae or draining sinuses, a cutaneous excision of the duct opening is recommended [18]. In the situation of the existence of residual tracts leading to the tonsillar fossa, beside the clear indication of extirpation of the tract itself, the necessity of ipsilateral tonsillectomy to prevent recurrence, is discussed [6, 19–22]. Overall, BCA-recurrence is stated up to 4% [23–25]. The aim of our analysis is to evaluate the need of ipsilateral tonsillectomy within the surgical treatment of BCA due to the recurrence rate in surgical treated BCA.

## Materials and Methods

The work has been reported in line with the STROCSS criteria in its updated version [26]. The trial has been registered under researchregistry7772, “The role of ipsilateral tonsillectomy in the extirpation of branchial cleft anomalies- A retrospective monocentric analysis over 13 years”.

### Institutional Review Board Review and Data Protection

The study is stated as exempt due to IRB approval and EU data protection regulations. Our retrospective chart review fits the exempt criteria. The research involves the collection of existing data, documents, records, pathological specimens or diagnostic specimens and the data is recorded in an anonymous manner such that subjects cannot be identified directly or through identifiers linked to the subject.

### Study Population

**Fig. 1:**
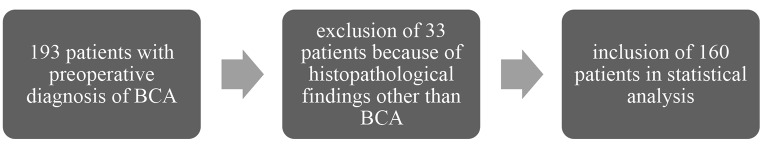
Study population and inclusion/exclusion algorithm

The study population was derived from the electronic database of all consecutive patients who admitted to the tertiary academic ENT department with the diagnosis of BCA between 2006 and 2020. (see Fig. 1 Study population and inclusion/exclusion algorithm)

### Data Collection and Statistical Analysis

We retrospectively evaluated patient charts, operation protocols and pathological reports. Relevant data were collected: gender, age, location of BCA (Robbin’s neck-level [27] and side), tonsillectomy yes/no, histopathological differentiation, duration of follow up and recurrence of BCA. Nominal scale data was described with frequency, ratio scale data over median and standard deviation. We performed a purely descriptive data analysis using the software IBM SPSS Statistics 26.

### Preoperative and Intraoperative Workup

Operative indication was based on anamneses, prior infections /neck swelling, clinical presentation and ultrasound or a MRI/CT to state the diagnosis of a BCA. In case of an existing fistula, excision of the skin duct and preparation along the tract with full extirpation was performed. Unilateral tonsillectomy was only performed, when a tract to the tonsillar fossa could be identified.

**Fig. 2:**
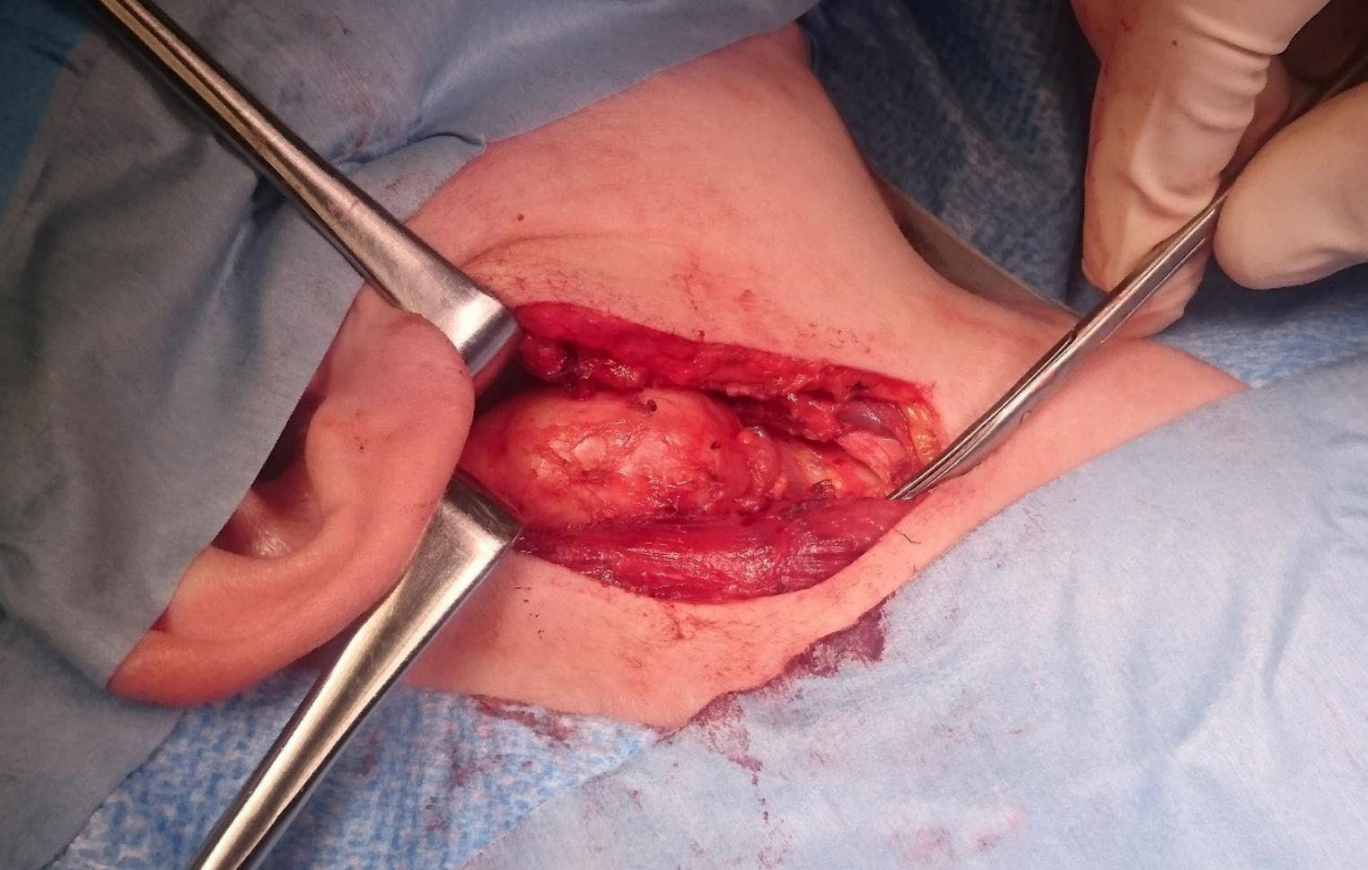
Intraoperative picture of lateral second branchial cleft cyst extirpation

**Fig. 3:**
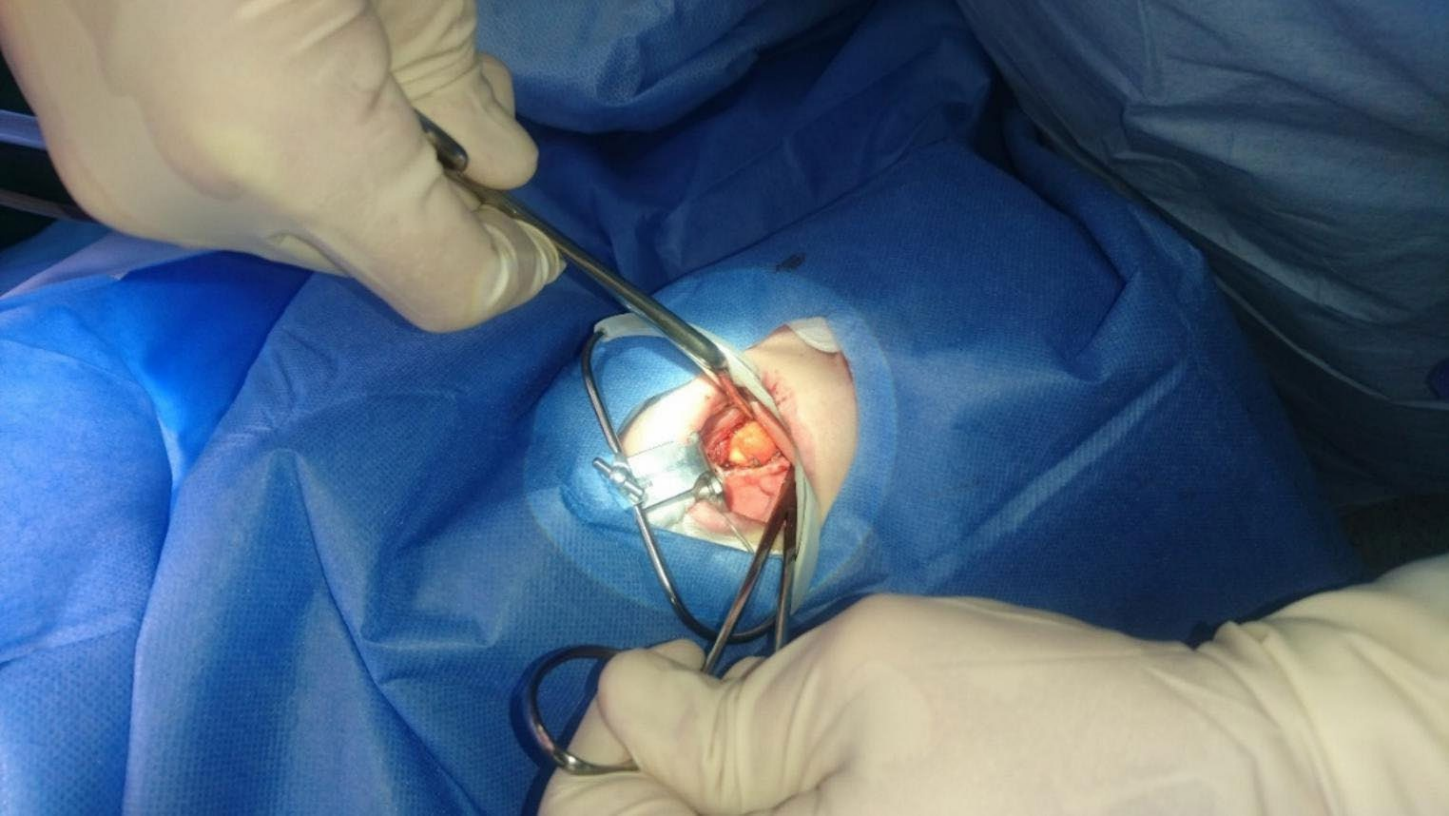
Intraoperative situs of a large second branchial cleft cyst in contact with the tonsillar fossa

By way of illustration, a second branchial cleft cyst in a female pre-tonsillectomized patient was operated over a modified neck dissection approach and transoral transection of the tonsillar region. The cyst had contact with the tonsillar fossa on the right side. (see Figs. 2 and 3)

## Results

The data of 160 patients (48.75% female; 51.25% male) included, comprised a median age of 35 years [3 M;83yrs]. The grouped age distribution is shown in Table 1. We could integrate 17 patients (10.6%), with a lateral branchial cleft fistulae, whereas the rest of 143 patients (89.4%) included, had a lateral branchial cleft cyst. Within the BCA, 54.37% were located on the left, and 45.63% located on the right side.

**Table 1:** Grouped age distribution in BCA

Age group	%
0 to 10 years	9.3% (N = 18)
10 to 20 years	17.1% (N = 33)
20 to 30 years	17.1% (N = 33)
30 to 40 years	13.5% (N = 26)
40 to 50 years	22.8% (N = 44)
50 to 60 years	9.3% (N = 18)
60 to 70 years	6.7% (N = 13)
70 to 80 years	2.6% (N = 5)
>80 years	1.6% (N = 3)
	∑=100%

Due to Robbin`s neck level [27] the most of BCA were located in the Level II (76.3%), followed by Level III (16.2%), Level I (2.7%), IV (2,0%) and V(1.4%), whereas 1.4% couldn’t be associated with a concrete Level.

When looking at the two BCA cohorts, within the fistula group, ipsilateral tonsillectomy was performed in 6 out of 17 patients (35.3%) and in 2 out of 143 patients within (1.4%) the branchial cleft cyst group. So, overall 8 out of 160 patients (5%) underwent ipsilateral simultaneous tonsillectomy.

Due to recurrence rate, we found relapses in 2 surgically treated patients within the branchial cleft cyst group (1.4%) and none within the branchial cleft fistula group, with a mean follow up of 31 months (26.9% readmission rate). Within the branchial cleft cyst group, 1 out of 2 patients underwent ipsilateral tonsillectomy, the other had no tonsillectomy. We could not prove a statistically significant difference in the recurrence-rate between the groups with or without tonsillectomy.

## Discussion

The aim of this work was to figure out, whether the recurrence-rate of BCA, where a tonsillectomy was performed, was lower than those BCA, where no tonsillectomy was performed. If this were the case, a simultaneous tonsillectomy during the extirpation of BCA would have been recommendable. In general, the recurrence rate of BCA after surgical excision is low. Within our data, in 2 out of 160 (1.25%) patients, we found BCA recurrency after surgical treatment. Both recurrency cases were evident within extirpated branchial cleft cysts and bilateral tonsillectomy in chronic tonsillitis (N = 143), further no recurrence occurred within the branchial cleft fistula group (N = 17).

In literature, BCA recurrency-rates ranges from 0 to 4% [15, 16, 25]. Due to the low recurrency-rate within our data, a consistent conclusion cannot be drawn. To statistically achieve that, at least 30 recurrences would have been necessary, corresponding to a total number of cases of 2150. Unfortunately, our study group was too small in order to achieve a significant statistical conclusion. Similarly, only 2 patients in the entire study group had recurrence following surgery (one of which underwent tonsillectomy) and so no consistent conclusions can be made in this regard. Despite that, it is important to note that 1/8 (12.5%) within the tonsillectomy group had recurrence in comparison to 1/152 (0.6%) in the non-tonsillectomy group. This tendency might strengthen the approach not to perform tonsillectomy regularly in BCA surgery.

The analysis of our data depicted, that tonsillectomies were performed in a reluctant manner. Tonsillectomies were solely performed in cases, where a level II fistula ended in the tonsillar fossa. This conservative behaviour is explained by the risk of postoperative haemorrhage, which is described between 1,9% and 6% after tonsillectomies [28–30]. Given the fact, that recurrence-rate of BCA is lower than the risk of postoperative bleeding in tonsillectomy, the standardized ipsilateral tonsillectomy should be avoided. In our opinion, even in an residual tract in contact to the tonsillar fossa, tonsillectomy can be avoided due to the risk/benefit ratio, in accord with other authors [6, 19–22].

Moreover, our data showed that 22.2% of patients that were initially suspected to have a BCA, in fact had a different diagnosis, that was of either benign or malignant histopathology. The reason for this was, that many of the patients were referred to the ENT department by either general practitioners or resident ENT specialists, that do not have the proper equipment to run necessary diagnostics. Further, preoperative diagnosis of cystic lateral neck masses can be crucial. Therefore, fine needle aspiration can be a sufficient tool to preoperatively detect potential malignancies [13]. Not to forget, that it is not uncommon to see unilateral tonsillar enlargement without the presence of a neck cysts. Further, preoperative diagnosis of an attachment between tonsil and the ipsilateral neck cyst can be complex.

A weakness of our study is a certain loss-of-follow-up, which cannot be numericized. The underlying cause is the chosen study design. We do not know, whether all treated patients in the tertiary academic ENT department were readmitted to the same hospital in case of BCA recurrency. Furthermore, there is a chance, that some patients still relapse in the future. These factors could explain, why the recurrence rate within our data is lower than in literature about this topic.

## Conclusion

The performance of an ipsilateral simultaneous tonsillectomy in the surgical workup of BCA cannot be recommended on the basis of our data due to the risk/benefit ratio.

## Electronic Supplementary Material

Below is the link to the electronic supplementary material.

Supplementary Material 1

## Statements and Declarations

The study is stated as exempt due to IRB approval and EU data protection regulations. Therefore the data acquisition and processing is excepted from ethics committee involvement. *The trial has been registered under researchregistry7772*.

## Level of Evidence

III (comparative retrospective monocentric study).
