# Supplementary material for: The Role of Ipsilateral Tonsillectomy in the Extirpation of Branchial Cleft Anomalies- A Retrospective Monocentric Analysis Over 13 Years
Source: Indian J Otolaryngol Head Neck Surg. 2023 Apr 21;75(3):1968–73. doi: 10.1007/s12070-023-03543-5 (PMC10447662; doi:10.1007/s12070-023-03543-5)
Supplement: Supplementary file 1 — Supplementary Material 1 [file 12070_2023_3543_MOESM1_ESM.docx]

**The role of ipsilateral tonsillectomy in the extirpation of branchial cleft anomalies- A retrospective monocentric analysis over 13 years-Revision letter**

Answers are being delivered in red.

COMMENTS TO THE AUTHOR:

Reviewer #1: Dear author as a reviewer I appreciate your BCA case series. There are few lacuna i can find in the submitted manuscript.

1)	Page 4 table 1.. comma needs to be replaced by point. Done

2)	Also in table 1 ( age distribution) BCA case sample is 160 but other than bca anomalies also included. So the cases meets the exclusion criteria means the HPE diagnosis other than BCA could have been excluded. No, we included 160 BCA anomalies in statistical analysis.

3)	Comma needs to be replaced by point in % at many places. Done.

4)	Page 8 line 29 (to perform) needs to be deleted .. thanks, done.

5)	Page 9 line 10 - at to be replaced by on. Done.

6)	In Branchial cleft cyst group 2 patients underwent tonsillectomy. The reason for tonsillectomy in cyst group is not elaborated. Correct, no see discussion (first paragraph).

7)	Also study groups patient lacks proper follow up so non recurrence is mostly assumption. Those patients who were not under follow up could have been excluded from the study . We could have excluded due to a loss of follow up. Even though we are not able to proof a low recurrence rate due to a partial follow up, we didn’t want to exclude patients under the assumption of a good treatment and perceived representation of patients in our clinic.

8)	Also there is lack of statistically significant correlation about recurrence and simultaneous tonsillectomies. Yes, we would have needed 2150 BCAs to deliver a statistically significant result. (see discussion)

Reviewer #2: It is not uncommon to see unilateral tonsillar enlargement without any neck cysts. It is very difficult to establish preoperatively the attachment between tonsil and the ipsilateral neck cyst. In BOR cases there is a possibility of a neck sinus/fistula tracking close to the ipsilateral tonsil from the skin surface and in such cases the extirpation of the ipsilateral tonsil may yield good outcomes and no recurrence of cyst or sinus. Totally true, but what can I do with this explicit statement? I tried to bring it in the discussion section.
